# Supplementary material for: Cooperative base pair melting by helicase and polymerase positioned one nucleotide from each other
Source: eLife. 2015 May 13;4:e06562. doi: 10.7554/eLife.06562 (PMC4460406; doi:10.7554/eLife.06562)
Supplement: Supplementary file 1. — DNA sequences used in unwinding and 2-aminopurine experiments. DOI: http://dx.doi.org/10.7554/eLife.06562.020 [file elife06562s001.pptx]

## Slide 1
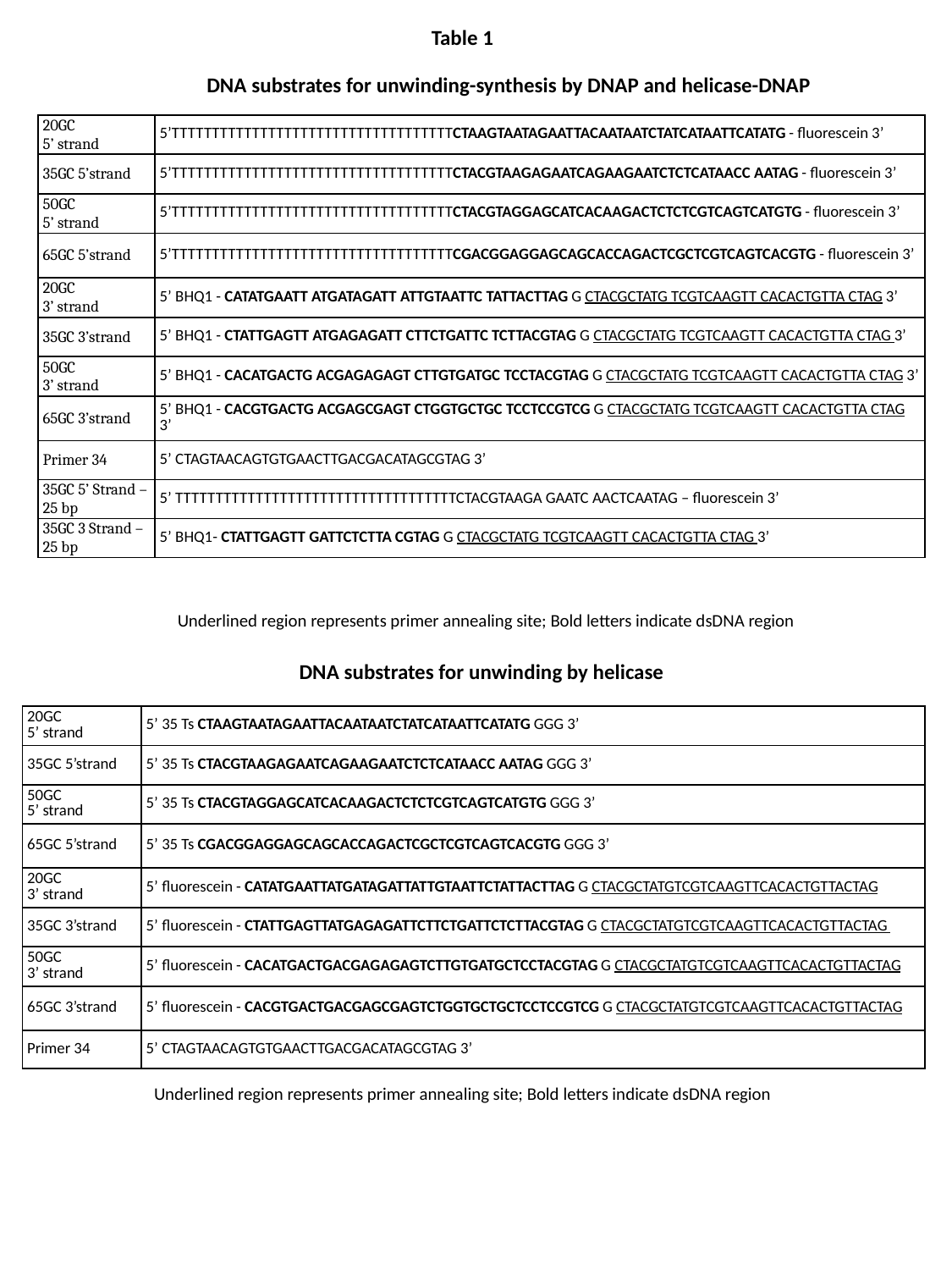

Table 1
DNA substrates for unwinding-synthesis by DNAP and helicase-DNAP
| 20GC 5’ strand | 5’TTTTTTTTTTTTTTTTTTTTTTTTTTTTTTTTTTTCTAAGTAATAGAATTACAATAATCTATCATAATTCATATG - fluorescein 3’ |
| --- | --- |
| 35GC 5’strand | 5’TTTTTTTTTTTTTTTTTTTTTTTTTTTTTTTTTTTCTACGTAAGAGAATCAGAAGAATCTCTCATAACC AATAG - fluorescein 3’ |
| 50GC 5’ strand | 5’TTTTTTTTTTTTTTTTTTTTTTTTTTTTTTTTTTTCTACGTAGGAGCATCACAAGACTCTCTCGTCAGTCATGTG - fluorescein 3’ |
| 65GC 5’strand | 5’TTTTTTTTTTTTTTTTTTTTTTTTTTTTTTTTTTTCGACGGAGGAGCAGCACCAGACTCGCTCGTCAGTCACGTG - fluorescein 3’ |
| 20GC 3’ strand | 5’ BHQ1 - CATATGAATT ATGATAGATT ATTGTAATTC TATTACTTAG G CTACGCTATG TCGTCAAGTT CACACTGTTA CTAG 3’ |
| 35GC 3’strand | 5’ BHQ1 - CTATTGAGTT ATGAGAGATT CTTCTGATTC TCTTACGTAG G CTACGCTATG TCGTCAAGTT CACACTGTTA CTAG 3’ |
| 50GC 3’ strand | 5’ BHQ1 - CACATGACTG ACGAGAGAGT CTTGTGATGC TCCTACGTAG G CTACGCTATG TCGTCAAGTT CACACTGTTA CTAG 3’ |
| 65GC 3’strand | 5’ BHQ1 - CACGTGACTG ACGAGCGAGT CTGGTGCTGC TCCTCCGTCG G CTACGCTATG TCGTCAAGTT CACACTGTTA CTAG 3’ |
| Primer 34 | 5’ CTAGTAACAGTGTGAACTTGACGACATAGCGTAG 3’ |
| 35GC 5’ Strand – 25 bp | 5’ TTTTTTTTTTTTTTTTTTTTTTTTTTTTTTTTTTTCTACGTAAGA GAATC AACTCAATAG – fluorescein 3’ |
| 35GC 3 Strand – 25 bp | 5’ BHQ1- CTATTGAGTT GATTCTCTTA CGTAG G CTACGCTATG TCGTCAAGTT CACACTGTTA CTAG 3’ |
Underlined region represents primer annealing site; Bold letters indicate dsDNA region
DNA substrates for unwinding by helicase
| 20GC 5’ strand | 5’ 35 Ts CTAAGTAATAGAATTACAATAATCTATCATAATTCATATG GGG 3’ |
| --- | --- |
| 35GC 5’strand | 5’ 35 Ts CTACGTAAGAGAATCAGAAGAATCTCTCATAACC AATAG GGG 3’ |
| 50GC 5’ strand | 5’ 35 Ts CTACGTAGGAGCATCACAAGACTCTCTCGTCAGTCATGTG GGG 3’ |
| 65GC 5’strand | 5’ 35 Ts CGACGGAGGAGCAGCACCAGACTCGCTCGTCAGTCACGTG GGG 3’ |
| 20GC 3’ strand | 5’ fluorescein - CATATGAATTATGATAGATTATTGTAATTCTATTACTTAG G CTACGCTATGTCGTCAAGTTCACACTGTTACTAG |
| 35GC 3’strand | 5’ fluorescein - CTATTGAGTTATGAGAGATTCTTCTGATTCTCTTACGTAG G CTACGCTATGTCGTCAAGTTCACACTGTTACTAG |
| 50GC 3’ strand | 5’ fluorescein - CACATGACTGACGAGAGAGTCTTGTGATGCTCCTACGTAG G CTACGCTATGTCGTCAAGTTCACACTGTTACTAG |
| 65GC 3’strand | 5’ fluorescein - CACGTGACTGACGAGCGAGTCTGGTGCTGCTCCTCCGTCG G CTACGCTATGTCGTCAAGTTCACACTGTTACTAG |
| Primer 34 | 5’ CTAGTAACAGTGTGAACTTGACGACATAGCGTAG 3’ |
Underlined region represents primer annealing site; Bold letters indicate dsDNA region

## Slide 2
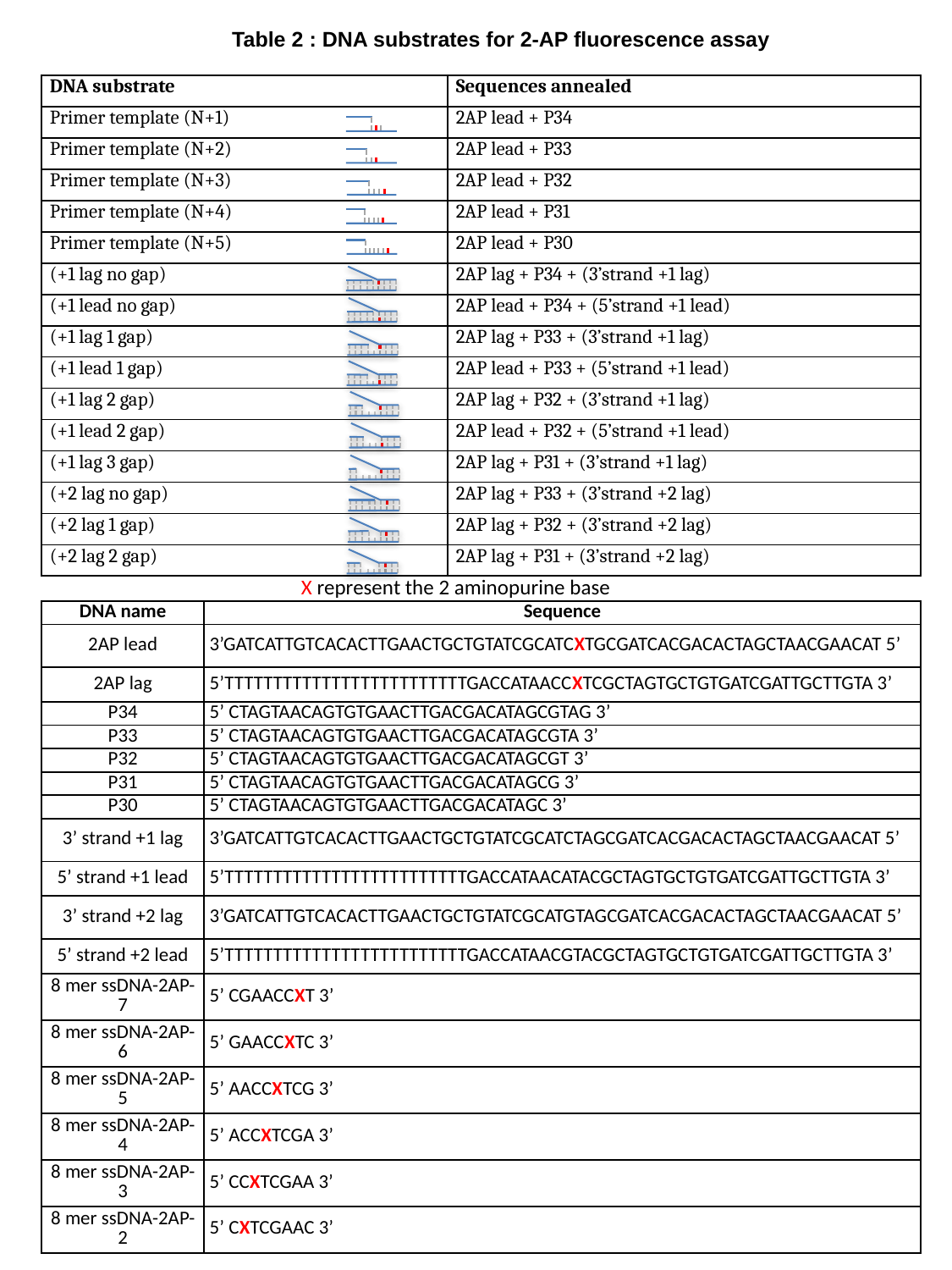

Table 2 : DNA substrates for 2-AP fluorescence assay
| DNA substrate | Sequences annealed |
| --- | --- |
| Primer template (N+1) | 2AP lead + P34 |
| Primer template (N+2) | 2AP lead + P33 |
| Primer template (N+3) | 2AP lead + P32 |
| Primer template (N+4) | 2AP lead + P31 |
| Primer template (N+5) | 2AP lead + P30 |
| (+1 lag no gap) | 2AP lag + P34 + (3’strand +1 lag) |
| (+1 lead no gap) | 2AP lead + P34 + (5’strand +1 lead) |
| (+1 lag 1 gap) | 2AP lag + P33 + (3’strand +1 lag) |
| (+1 lead 1 gap) | 2AP lead + P33 + (5’strand +1 lead) |
| (+1 lag 2 gap) | 2AP lag + P32 + (3’strand +1 lag) |
| (+1 lead 2 gap) | 2AP lead + P32 + (5’strand +1 lead) |
| (+1 lag 3 gap) | 2AP lag + P31 + (3’strand +1 lag) |
| (+2 lag no gap) | 2AP lag + P33 + (3’strand +2 lag) |
| (+2 lag 1 gap) | 2AP lag + P32 + (3’strand +2 lag) |
| (+2 lag 2 gap) | 2AP lag + P31 + (3’strand +2 lag) |
X represent the 2 aminopurine base
| DNA name | Sequence |
| --- | --- |
| 2AP lead | 3’GATCATTGTCACACTTGAACTGCTGTATCGCATCXTGCGATCACGACACTAGCTAACGAACAT 5’ |
| 2AP lag | 5’TTTTTTTTTTTTTTTTTTTTTTTTTGACCATAACCXTCGCTAGTGCTGTGATCGATTGCTTGTA 3’ |
| P34 | 5’ CTAGTAACAGTGTGAACTTGACGACATAGCGTAG 3’ |
| P33 | 5’ CTAGTAACAGTGTGAACTTGACGACATAGCGTA 3’ |
| P32 | 5’ CTAGTAACAGTGTGAACTTGACGACATAGCGT 3’ |
| P31 | 5’ CTAGTAACAGTGTGAACTTGACGACATAGCG 3’ |
| P30 | 5’ CTAGTAACAGTGTGAACTTGACGACATAGC 3’ |
| 3’ strand +1 lag | 3’GATCATTGTCACACTTGAACTGCTGTATCGCATCTAGCGATCACGACACTAGCTAACGAACAT 5’ |
| 5’ strand +1 lead | 5’TTTTTTTTTTTTTTTTTTTTTTTTTGACCATAACATACGCTAGTGCTGTGATCGATTGCTTGTA 3’ |
| 3’ strand +2 lag | 3’GATCATTGTCACACTTGAACTGCTGTATCGCATGTAGCGATCACGACACTAGCTAACGAACAT 5’ |
| 5’ strand +2 lead | 5’TTTTTTTTTTTTTTTTTTTTTTTTTGACCATAACGTACGCTAGTGCTGTGATCGATTGCTTGTA 3’ |
| 8 mer ssDNA-2AP-7 | 5’ CGAACCXT 3’ |
| 8 mer ssDNA-2AP-6 | 5’ GAACCXTC 3’ |
| 8 mer ssDNA-2AP-5 | 5’ AACCXTCG 3’ |
| 8 mer ssDNA-2AP-4 | 5’ ACCXTCGA 3’ |
| 8 mer ssDNA-2AP-3 | 5’ CCXTCGAA 3’ |
| 8 mer ssDNA-2AP-2 | 5’ CXTCGAAC 3’ |
